# Possibilities of Useful Use of Glued Wooden Construction Residues

**DOI:** 10.3390/ma14154106

**Published:** 2021-07-23

**Authors:** Peteris Resnais, Aldis Grekis, Matiss Keivs, Baiba Gaujena

**Affiliations:** 1 “Funduss” Ltd., Tiltakrogs, Ieriki, LV-4139 Amatas Novads, Latvia; peteris@funduss.lv; 2 Institute of Heat, Gas and Water Technology, Riga Technical University, LV-1048 Riga, Latvia; m.keivs@rtu.lv; 3 Institute of Building Production, Riga Technical University, LV-1048 Riga, Latvia; baiba.gaujena@rtu.lv

**Keywords:** construction, building, timber structures, CLT panels

## Abstract

People have erected buildings with the use of timber structures for a long time. The uses of timber constructions are very diverse—it is used for the production of exterior wall and roof constructions, window frames and doors, and it is used for dry as well as wet premises. Scandinavian countries have extremely vast experience of using timber structures. Latvia has a rather extensive timber processing and timber structure manufacturing sector. Many companies are involved in timber processing, however, to enable even more extensive use of timber structures, environmental and technically economic requirements of contemporary building must be taken into consideration. Environmental requirements for timber structures provide certain advantages in comparison to other building materials, but technically economic requirements are very important as well. The development of manufacturing of glued constructions and research of production processes of these constructions allows one to find solutions for the reduction in the cost of timber structures, and the results of such research can ensure significant development of the use of timber structures in building, as well as reduce total construction costs. The basic objective of the study is to investigate the residual materials arising as a result of processing cross-laminated timber constructions (CLT panels), material generated as a result of high levels of construction production, and research of the opportunities to reprocess the residual materials generated as a result of laminated timber structure manufacturing into materials suitable for production of building constructions. The majority of CLT panels are manufactured using 20, 30 and 40 mm thick boards, and, during the panel manufacturing process, there are various standard thicknesses of panels, for example, 60, 80, 100, 120, 140, 160 mm, etc. Various layers are used for the creation of various thicknesses depending on the necessary technical properties. Various arrangements of the thickness of a single panel will cause different structural and physical behaviour (i.e., impact of changes in moisture, fire resistance, etc.). During the research and for the purposes of testing of CLT panels, only residues with equal types and thicknesses of lamellae were selected. Two main purposes were included in the panel testing process: (1) Comparison of technical performance of the residues of CLT panels with the classic CLT panel. Curve strength and tensile strength tests were performed in accordance with LVS EN standards (LVS EN 16351: 2016 and LVS EN 408 + A1: 2012). All the samples were prepared according to the LVS EN standards. (2) To assess the impact of two resins (melamine urea formaldehyde (MUF) and polyurethane (PU)), widely used in industry, on structural properties of recycled CLT material. Results of the research show that recycling residues of glued wooden constructions may lead to good results, and manufacturing residues of CLT panels may be successfully used in construction and for the reduction in CLT panel manufacturing costs.

## 1. Introduction

The principal objective of the study is to research the manufacturing process of cross-laminated timber constructions (hereinafter CLT panels) and to optimise the residues generated as a result of CLT panel manufacturing to create a material suitable for the building of large constructions. The aim is to research the opportunities of reprocessing the residues arising as a result of the production of laminated timber structures into materials suitable for the manufacturing of building constructions. The potential solution is the formatting of the residues and increasing the dimensions by using large finger joints and construction class glues. In other research [1,2,3] on timber construction, hydrothermal performances and thermal properties of wooden constructions were mostly studied, which are no less important than the strength of different types of constructions. It is necessary to take into account the connection details with the foundation if the bearing vertical constructions are made from wood, and also if the boundary constructions are made from lightweight materials, so one cannot separate these studies because they work together to ensure safe constructions.

However, cross-laminated timber constructions in the form of CLT panels are a modern building material that possess multiple positive properties—natural, environmentally friendly, sustainable and safe. The panels are made of fir tree planks that are laminated in orthogonal layers by using formaldehyde-free polyurethane glue, which results in a very durable and safe building material.

CLT panels are extensively used in construction—for the construction of load-bearing walls, floors, roof constructions and other constructions of different types. The panels can also be used for the production of stairs, furniture and other materials.

CLT panels conform to the C 24 class and are lighter than other construction materials in terms of weight, e.g., lightweight concrete and concrete blocks, which increases the competitiveness of the panels against other materials, since they are easier to transport and assemble. The fact that CLT panels have excellent fire safety properties is especially interesting; CLT, being a solid wood construction, is very slow to heat up and has better fire safety properties than metal and concrete.

Achieving the economic use of the raw material and efficiency is a common problem in the production of glued timber structures (glued laminated beams, CLT). During the manufacturing process, especially when using standard size glue-laminated beams and CLT panels, the production residue amounts to an average of 5–10% in the form of glued timber that, due to inappropriate dimensions, is not used for the intended purpose—building constructions.

Residue of CLT panel construction occurs while sawing the required panel sizes, cutting door and window frames and cutting other openings.

Currently, there are three main directions for the utilisation of glued timber structure residues:

- The dominant approach involves the use of glued timber residues as fuel. The main problem with this solution is the fact that some of the residue (CLT) must be reduced to smaller dimensions prior to the production of woodchips due to technical limitations and the end product—woodchips have relatively low added value.
- Reprocessing of the residues into transportation and packaging materials. Due to highly variable dimensions of the materials, it is a laborious method resulting in a low-quality outcome.
- Storage of residues for use in future production projects. The approach is not popular, since, in most cases, the dimensions of the residue are smaller than required for the manufacturing of building constructions, the storage thereof consumes a lot of space and inventory and the sorting and selection of materials for use in future construction projects is a resource- and time-consuming process.

Although there is research [4] on wooden multi-span beams with steel reinforcement and proves that the high durability of wooden structures with glued reinforcement increases the strength, with a similar method in this study, research was conducted on new solutions and new technology that would enable the reprocessing of the residues arising as a result of the production of glued timber structures into large raw materials suitable for the production of building constructions. The solution for reaching the target is the formatting of the residues and increasing the dimensions to dimensions that can be effectively reprocessed by using large finger joints and construction class glues.

The current research area worldwide has not included the impact of the quantity of finger joints on the technical performance of the recycled CLT. Likewise, there are no analogous solutions for finger joints and, furthermore, there is a small amount of research on this topic. At the moment, this research is at the initial stage, and many additional measures are provided to justify the technologies used in this research in the future.

Within the research, it was planned to study the possibility to recycle residues of the CLT panels available on the market. However, it was determined during the technology testing process that the smallest size of residues to be recycled should be limited for the purposes of manufacturing performance targets. Both economic and technical aspects concerning the quantity of finger joints are the subjects of further research, since a wider area regarding both the availability and final use of residues must be considered.

## 2. Materials and Methods

**Figure 1:**
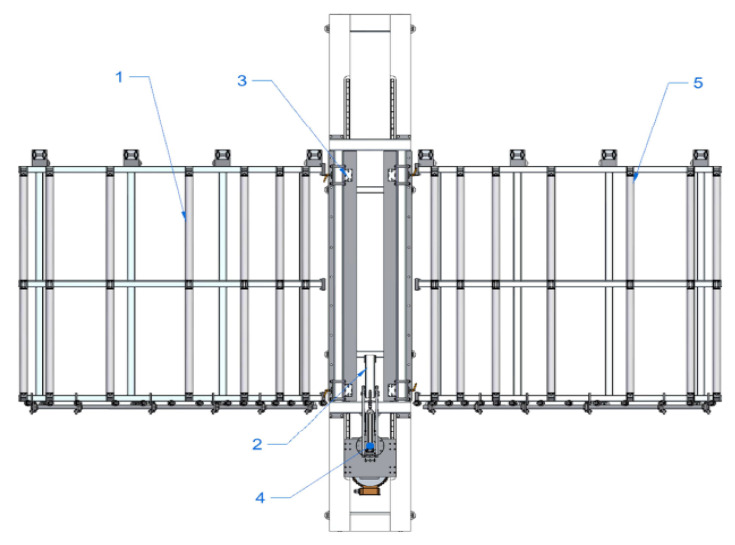
Processing scheme of CLT finger joint mill (1—Assembly table, 2—Removable lock point, 3—Compressing clamps, 4—Milling head with carriage, sawdust removal system, 5—Removal table).

A new device with a semi-automatic method of finger joint insertion is used for the use of the residues of glued timber structures for the production of new building constructions. This is a device of a completely new design without analogues in other countries (see Figure 1).

Sequence of works for the processing of a CLT panel blank and operation of the device:

1. The residues are sorted and formatted to create blanks of similar dimensions;
2. Milling of finger joints:

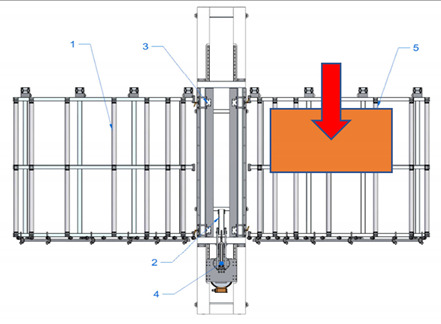
Assembly table,Removable lock point,Compressing clamps,Milling head with carriage, sawdust removal system,Removal table.The residues are sorted and formatted to created blanks of similar dimensions.
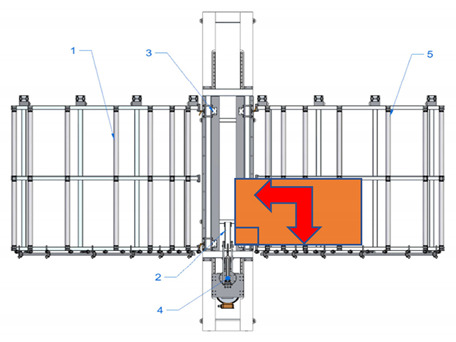
Assembly table,Removable lock point,Compressing clamps,Milling head with carriage, sawdust removal system,Removal table.b.The residue piece is manually aligned with two lock points: side and tip, forming a straight angle between the longitudinal side of the panel and trajectory of movement of the router head.
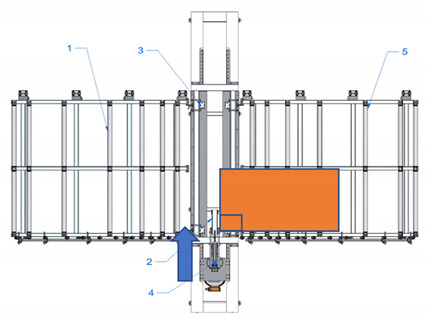
Assembly table,Removable lock point,Compressing clamps,Milling head with carriage, sawdust removal system,Removal table.c.The residue piece is pneumatically fixed in position from above.d.Finger joint is milled into one end of the residue piece by moving the router head.
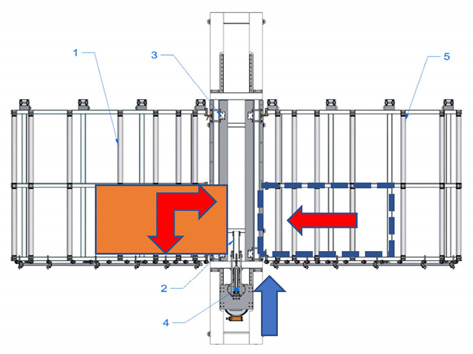
Assembly table,Removable lock point,Compressing clamps,Milling head with carriage, sawdust removal system,Removal table.eThe residue piece is released.f.The residue piece is removed for pressing or it can be pushed through the milling area to the removal table for secondary profiling. The removal table is ½ of the finger joint profile height.g.The second (or the same residue piece if it is necessary to have finger joints on both ends of the panel) residue piece is manually aligned with two lock points.
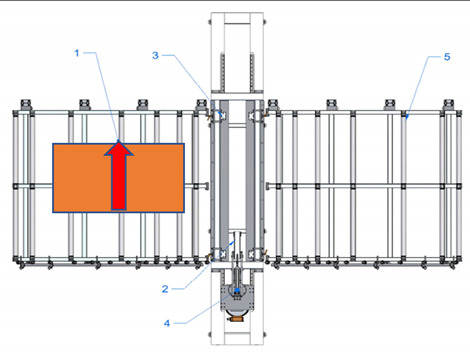
Assembly table,Removable lock point,Compressing clamps,Milling head with carriage, sawdust removal system,Removal table.h.The residue piece is compressed from above and the finger joint is milled into the tip of the second residue piece (or the same piece from the other end), thus creating the finished secondary blank.i.The panel is released and removed from the removal table.
3. Pressing of the panel.

The blanks are placed on the press, the glue is applied on the finger joint and the secondary blanks are compressed and held for a period of time, as a result of which the prepared blank for the preparation of a CLT panel is ready. To efficiently achieve the required dimensions of the material (i.e., min. 15–20 mg) for a CNC intended for the processing of CLT, the process needs to be repeated by increasing the dimensions and creating finger joints orthogonally relative to the joints inserted in the first cycle.

As a result, by using a finger joint CLT mill, samples were obtained and the following actions were performed:

- the problems associated with the manufacturing of the product were researched, the manufacturing results of the product were improved and the production of test samples of the product was performed;
- the testing of physical and mechanical properties of the product samples manufactured during the development of the technology was performed to assess the effect of various glues on the physical and mechanical properties of the product and to assess manufacturing processes;
- the determining of physical and mechanical characteristics of the product and the comparison thereof with the products manufactured in accordance with classical technology were performed.

**Figure 2:**
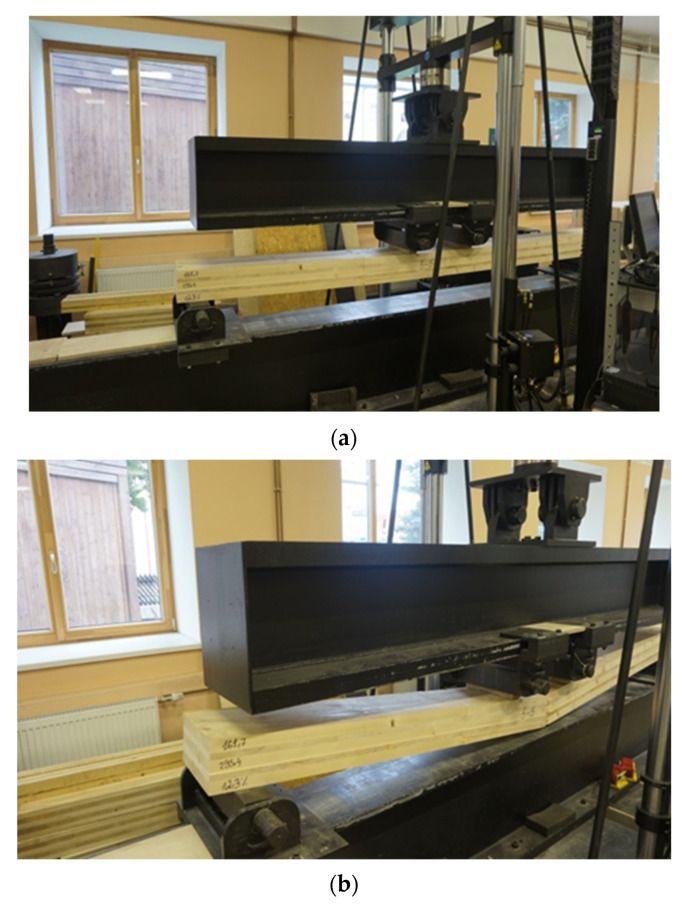
Measuring equipment for determining flexural strength: (**a**) view; (**b**) close view.

The borderline flexural strength limits of the CLT panel residue samples were determined on the measuring equipment (see Figure 2a,b) of Forest and Wood Products Research and Development Institute Ltd (Dobeles street 41, Jelgavas city, Latvia).

## 3. Results

As a result of the study, samples made from the residues of the manufacturing of glued timber structures (CLT panels) were obtained by means of using a new device with a semi-automatic development of a finger joint.

To test the technical characteristics of the experimental CLT panels, tests were performed in accordance with [5,6,7] and [8,9,10,11,12,13].

During the measurements for the determining of borderline flexural strength of the samples made of CLT panel production residues, the following results were obtained:

1. Samples of group No. 1. Average size of the sample 101.59 × 296.94 mm^2^ (see Table 1).

**Figure 3:**
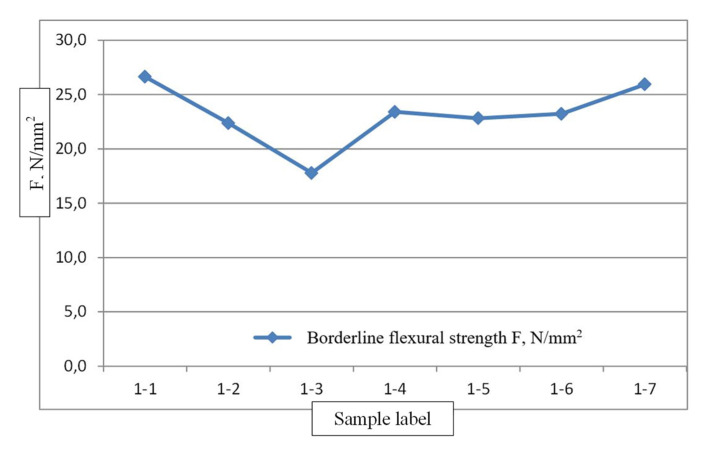
Borderline flexural strength N/mm^2^ measurement graph for samples of group No. 1.

The average value of borderline flexural strength of samples of group No. 1 is 23.2 N/mm^2^ (see Figure 3).

2. Samples of group No. 2. Average size of the sample 161.30 × 298.20 mm^2^ (see Table 2).

**Figure 4:**
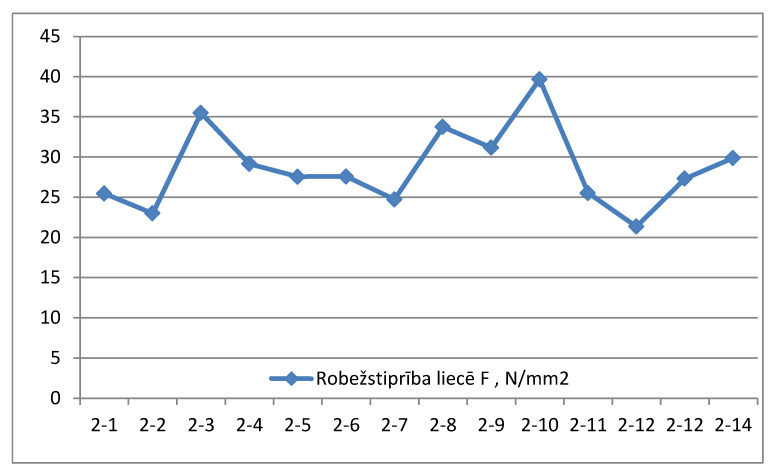
Borderline flexural strength N/mm^2^ measurement graph for samples of group No. 2.

The average value of borderline flexural strength of samples of group No. 2 is 28.7 N/mm^2^ (see Figure 4).

3. Samples of group No. 3. Average size of the sample 297.44 × 101.60 mm^2^ (see Table 3).

**Figure 5:**
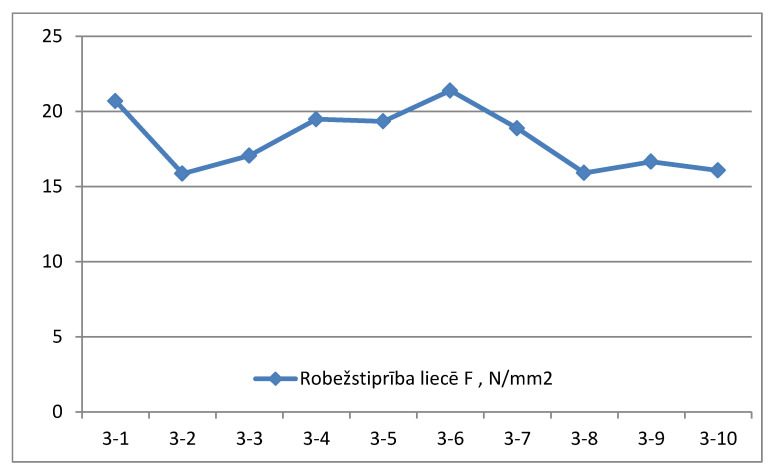
Borderline flexural strength N/mm^2^ measurement graph for samples of group No. 3.

The average value of borderline flexural strength of samples of group No. 3 is 18.1 N/mm^2^ (see Figure 5).

The summary of the characteristics of samples made from the residues of glued timber structures (CLT panels) is shown in Table 4.

**Figure 6:**
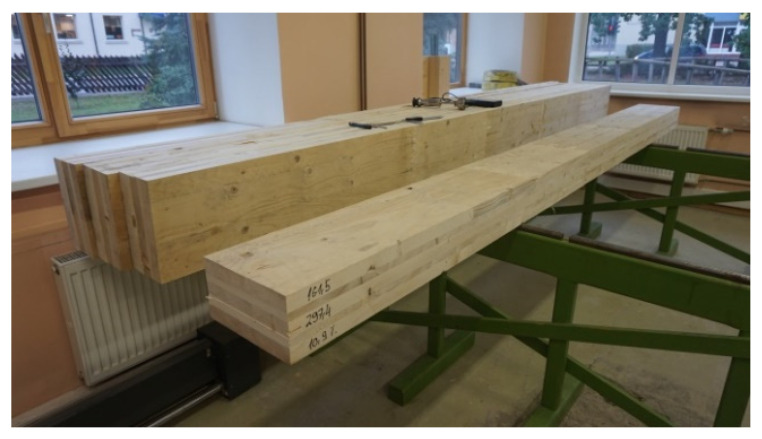
Preparation of sample and performance of measurements (sample dimension and relative humidity measurements).

**Figure 7:**
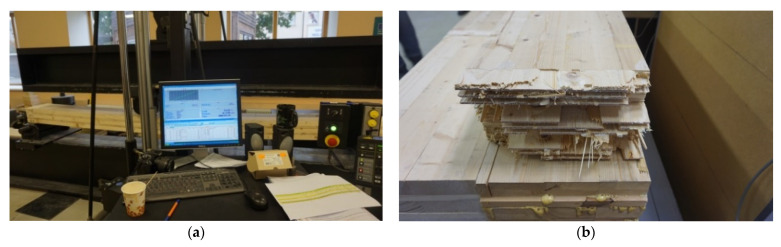
(**a**) Performance of sample measurements (**b**) Analysis of the sample.

Preparation of the sample and performance of measurements (sample dimension and relative humidity measurements) are shown in Figure 6 and performance of sample measurements and analysis of the sample are shown in Figure 7.

## 4. Conclusions

The results of the study will enable the optimisation of the manufacturing process by reprocessing the residues arising during CLT processing into material suitable for the production of large constructions, thus ensuring economic use of the material as well as manufacturing efficiency.

The created device with a semi-automatic finger joint insertion method is a unique device that will enable a considerable increase in the development of CLT panels and reduce the cost of panels. Nevertheless, it must be considered that time is required to improve the operation and efficiency of the device and to ensure stable and precise technical parameters of the production residue of CLT panels. Additional research on the improvement of the application of glue and treatment processes is required.

CLT panels are manufactured using 20, 30 and 40 mm boards, which allow one to obtain various standard thicknesses of panels, e.g., 60, 80, 100, 120, 140, 160 mm, etc. Various layers of layers are used for the creation of various thicknesses depending on the necessary technical properties, for example, structural, fire, visual properties or economic aspects. Therefore, residues of CLT panels with similar types of lamellae were selected for testing.

The results of measurements of the borderline flexural strength of samples made of CLT panel production residues show that samples of group No. 2 (average dimensions of the sample 161.30 mm × 298.20 mm) reach C 27 requirements of flexural strength (average borderline flexural strength 28.7 N/mm^2^); samples of group No. 1 (average dimensions of the sample 101.59 mm × 296.94 mm) reach C 22 requirements of flexural strength (average borderline flexural strength 23.2 N/mm^2^); while samples of group No. 3 (average dimensions of the sample 297.44 mm × 101.60 mm) reach C 18 requirements of flexural strength (average borderline flexural strength 18.1 N/mm^2^).

The results of borderline flexural strength measurements of samples obtained from CLT panel production residues prove that the obtained results are sufficient for the successful use of CLT panel production residues in construction, thus reducing the production costs of CLT panels. Samples used in measurements and the results of measurements demonstrate that the performance of additional research and improvements in the efficiency of material production may lead to results that are equivalent to the technical parameters of new CLT panels., i.e., having the strength class of C 24. Currently, samples of group No. 2 are capable of meeting the set flexural strength requirements.

The upgrading of material production technology and the achievement of predictable results in research works may enable the use of CLT panels made from production residues for the construction of bearing structures of buildings and calculations thereof in accordance with [14,15,16].

The recycling of CLT panel production residues for construction instead of fuel transportation and packaging or storage needs will provide a significant environmental and economic effect in the use of glued timber constructions in the building sector. The beneficiaries from the use of recycled CLT panels would be the producers of panels as well as builders and owners of the buildings.

Elements of CLT construction are cut from square-shaped main panels, all the window and door openings, geometric cuts and differences in dimensions of CLT construction elements, resulting in a significant quantity of residue material. The majority of this material is not useful for construction due to the dimensions.

Unfortunately, residues of CLT panels are paid for by the end user (owner of the building). The aim of the technology is the reduction in the gap and manufacturing of byproducts with the highest possible value. The final goal would be to create a recycled CLT product, whose technical indicators are not different from the standard CLT to make it usable and sellable within the same price range.

## Figures and Tables

**Table 1:** Characteristic values of borderline flexural strength, group 1 (load applied on the widest face).

Sample Label	Height b, mm	Width h, mm	Moisture W, %	Maximum Load Pmax, N	Borderline Flexural Strength F, N/mm^2^	Time to Maximal Load, s
1-1	101.70	302.10	11.3	30,833	26.6	507
1-2	101.90	301.20	10.1	31,105	22.4	257
1-3	101.30	289.50	10.4	19,563	17.8	217
1-4	101.40	292.40	10.5	26,065	23.4	255
1-5	101.50	299.40	10.8	26,069	22.8	292
1-6	101.40	298.40	10.5	26,392	23.2	280
1-7	101.90	295.60	10.5	33,183	25.9	325
Mean value			10.6	27,601	23.2	305
Standard deviation			0.4	4558	2.9	
Coefficient of variation			3.6	16.5	12.4	
5% quantile parameter value					19.2	

**Table 2:** Characteristic values of borderline flexural strength, group 2 (load applied on the widest face).

Sample Label	Height b, mm	Width h, mm	Moisture W, %	Maximum Load Pmax, N	Borderline Flexural Strength F, N/mm^2^	Time to Maximal Load, s
2-1	162.2	299.8	13.3	46,497	25.5	433
2-2	162.4	297.5	13.1	41,770	23.0	280
2-3	160.8	297.4	12.1	63,156	35.5	416
2-4	160.1	299.4	12.5	51,768	29.1	335
2-5	161.7	299.4	12.3	49,904	27.5	322
2-6	161.2	296.5	11.9	49,183	27.6	357
2-7	161.5	297.4	10.9	44,382	24.7	319
2-8	160.7	296.3	11.7	59,756	33.7	376
2-9	161.4	297.5	12.0	55,899	31.2	364
2-10	161.0	299.3	12.7	71,221	39.7	489
2-11	162.1	307.4	11.0	47,731	25.5	316
2-12	162.1	301.9	13.2	75,280	21.4	611
2-12	160.8	287.1	12.7	46,936	27.3	321
2-14	160.5	298.1	12.6	53,115	29.9	344
Mean value			12.3	54,043	28.7	377
Standard deviation			0.7	10,013	5.0	
Coefficient of variation			6.1	18.5	17.5	
5% quantile parameter value					22.4	

**Table 3:** Characteristic values of borderline flexural strength. Group 3 (load applied on the narrowest face).

Sample Label	Height b, mm	Width h, mm	Moisture W, %	Maximum Load Pmax, N	Borderline Flexural Strength F, N/mm^2^	Time to Maximal Load, s
3-1	292.00	101.00	12.0	36.002	20.7	333
3-2	298.50	101.30	10.9	28.906	15.9	313
3-3	297.20	101.50	10.6	30.892	17.1	360
3-4	300.30	102.00	10.2	36.204	19.5	377
3-5	302.10	101.80	10.5	36.301	19.3	353
3-6	296.40	101.30	9.8	38.455	21.4	411
3-7	288.20	101.80	10.8	32.238	18.9	361
3-8	300.20	101.90	10.2	29.512	15.9	287
3-9	297.40	101.50	9.7	30.201	16.7	296
3-10	302.10	101.90	10.1	30.201	16.1	298
Mean value			10.5	32.891	18.1	339
Standard deviation			0.7	3.487	2.1	
Coefficient of variation			6.3	10.6	11.4	
5% quantile parameter value					15.9	

**Table 4:** Summary of sample borderline flexural strength parameter (loaded on the narrowest face).

Sample Label	Maximum Load Pmax, N	Borderline Flexural Strength F, N/mm^2^	Time to Maximal Load, s
1	27,601	23.2	305
2	54,043	28.7	377
3	32,891	18.1	339

## Data Availability

The data presented in this study are available on reasonable request from the corresponding author.
